# Rupture of sinus of Valsalva aneurysm: a case report in a child

**DOI:** 10.1186/s12872-022-02603-y

**Published:** 2022-04-09

**Authors:** Kunfeng Jiang, Jingyu Chen, Xu Zhu, Huan Xiao, Tingting Ran, Yi Tang, Xiaojuan Ji

**Affiliations:** 1 Department of Ultrasound, Children’s Hospital of Chongqing Medical University, Ministry of Education Key Laboratory of Child Development and Disorders, National Clinical and Research Center of Child Health and Disorders, China International Science and Technology Cooperation Base of Child Development and Critical Disorders, Chongqing Engineering Research Center of Stem Cell Therapy, Chongqing, 400016 China; 2 Department of Ultrasound, Chongqing General Hospital, Chongqing, China

**Keywords:** Sinus of Valsalva aneurysm, Acute heart failure, Echocardiography, Percutaneous closure

## Abstract

**Background:**

Sinus of Valsalva aneurysm (SVA) is a rare congenital disease that can cause severe clinical presentations when the aneurysm ruptures. Here, we report a rare case of a noncoronary sinus of Valsalva aneurysm with rupture into the right atrium.

**Case presentation:**

A 14-year-old Chinese female patient presented viral myocarditis with acute heart failure at the local hospital, and she was finally diagnosed with a noncoronary sinus Valsalva aneurysm with rupture into the right atrium by digital subtraction angiography with cardiac catheterization angiography and echocardiography at our hospital (Children’s Hospital of Chongqing Medical University). Percutaneous closure intervention was performed shortly after her diagnosis, and the patient showed good functional recovery.

**Conclusions:**

We report a case of ruptured sinus of Valsalva aneurysm successfully treated by percutaneous closure, which is an excellent alternative treatment.

## Background

Sinus of Valsalva aneurysm (SVA) is a rare cardiac anomaly of the coronary sinuses caused by the absence of elastic tissue between the aorta and the annulus fibrosus [1]. The incidence of SVA accounts for 0.1–3.5% of congenital heart disease cases [2]. The mechanism of SVA involves deficiencies of muscle and elastic fibres in the middle layer, which progress into an aneurysm in the weakened area [3]. Patients who have SVA remain asymptomatic until one of the coronary sinuses ruptures into the cardiac chamber. According to previous reports, rupture of SVA usually occurs in adults, and the male:female sex ratio is 2–4:1 [4]. Sinus of Valsalva aneurysm most frequently originates from the right coronary sinus (70–90%), followed by the noncoronary sinus (10–25%) and, rarely, the left sinus (< 5%) [5]. SVA usually ruptures into the right ventricle (RV), then the right atrium (RA), and finally the left ventricle (LV) [6]. In this report, we present a rare case of noncoronary SVA rupture into the RA with acute heart failure.

## Case presentation

A 14-year-old girl presented to the local hospital with vomiting, abdominal pain, and diarrhoea and was initially diagnosed with viral myocarditis, acute heart failure, and pneumonia. She was transferred to our department because her clinical symptoms had progressed to shortness of breath, chest tightness, fatigue, decreased activity, and white, foamy sputum. On physical examination, the girl was 49 kg in weight and 151 cm in height, her blood pressure was 127/71 mm·Hg, her heart rate was 125 bpm, her respiratory rate was 30 breaths per minute, and her transcutaneous oxygen saturation (SpO_2_) was 96%. Cardiac examination showed a normal S1 and S2 pulse, but a holosystolic murmur (grade 3/6) at the second left intercostal space.

**Fig. 1:**
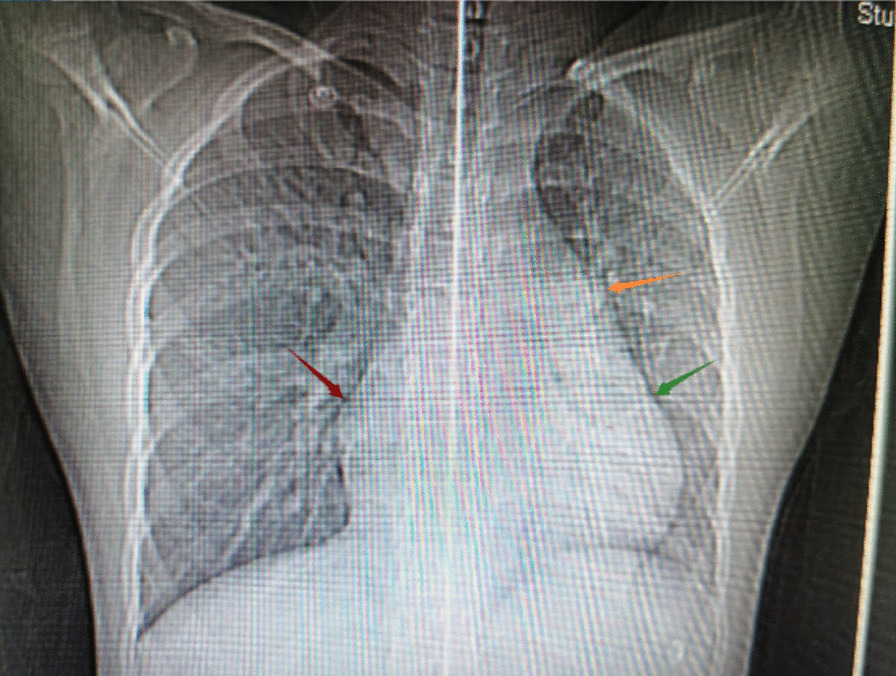
Chest X-ray of the patient. The yellow arrow represents the widening of the pulmonary artery segment; the red arrow represents the enlargement of the RA; and the green arrow represents the enlargement of the RV

**Fig. 2:**
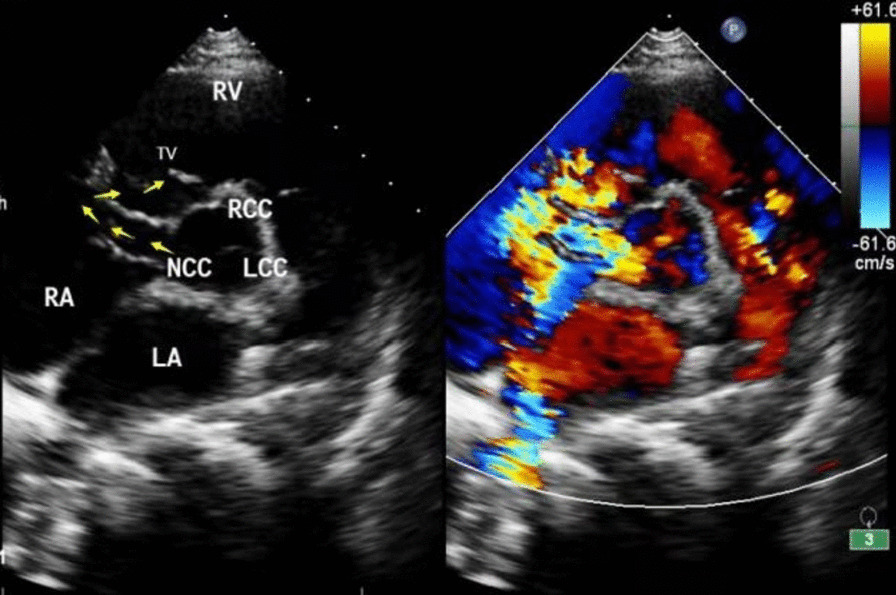
Echocardiography with colour Doppler showing a high-velocity multicoloured (aliasing) mosaic of blood flow from the NCC to RA. The yellow arrow represents the shunt. NCS: noncoronary sinus; RCS: right coronary sinus; LCS: left coronary sinus; RA: right atrium; RV, right ventricle; LA: left atrium; TV, tricuspid valve

Electrocardiogram revealed sinus tachycardia and changes in the T wave. Chest X-ray displayed an enlarged cardiac silhouette (Fig. 1). A transthoracic echocardiogram (mid-aortic valve short-axis view demonstrated enlargement of the aortic sinus and a turbulent colour flow from the noncoronary sinus rupturing into the RA above the tricuspid valve (Fig. 2). In addition, transthoracic echocardiography also revealed enlargement of the cardiac chamber, aortic valve regurgitation (AR, mild, 2.5 mm), tricuspid regurgitation (TR, moderate, 3.6 mm), pulmonary hypertension (PH, moderate; flow rate of pulmonary valve regurgitation, 2.12 m/s; PASP≈53 mm·Hg), hydropericardium, left ventricular ejection fraction (LVEF) of 71% and left ventricular fractional shortening (LVFS) of 41%. Furthermore, transthoracic echocardiography was also used to measure the aortic dimensions, which are shown in Table 1.

**Table 1:** Comparison of the aortic dimensions before and after the percutaneous closure intervention

Time	Aortic dimensions (mm)				
	Annulus	Valsalva sinuses	Sinotubular junction	Distal ascending aorta	z values of the annulus
Preoperative	29	37	27	27	5.6
Postoperative Day 3	27	34	25	27	4.5
Postoperative Month 1	22	31	25	27	1.76

**Fig. 3:**
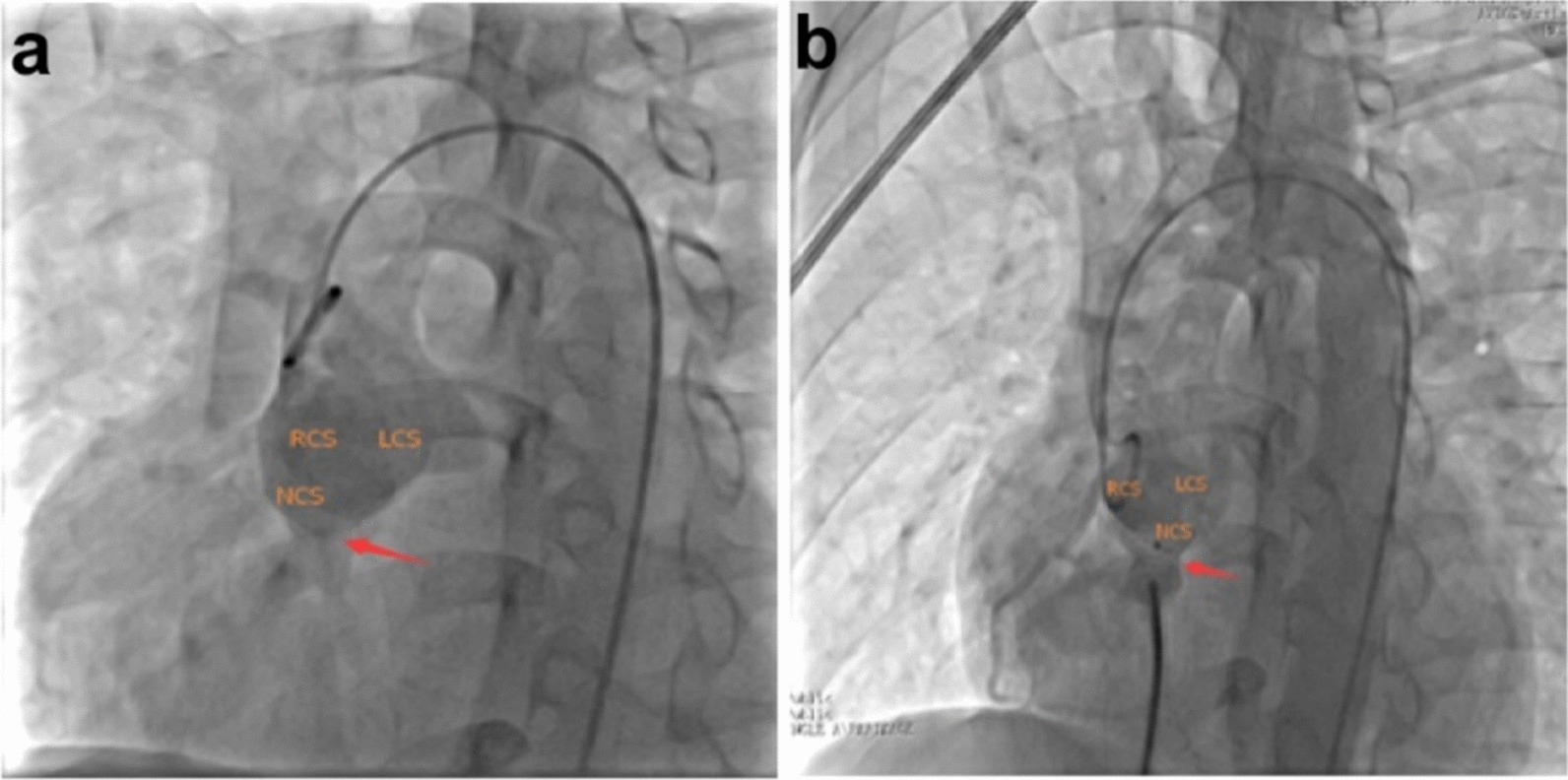
**a** Long axial oblique views showing a noncoronary sinus aneurysm rupturing into the right atrium (yellow arrow); **b** complete occlusion after device implantation (yellow arrow: rupture site or device). LCS, left coronary sinus; NCS, noncoronary sinus; RCS, right coronary sinus

**Fig. 4:**
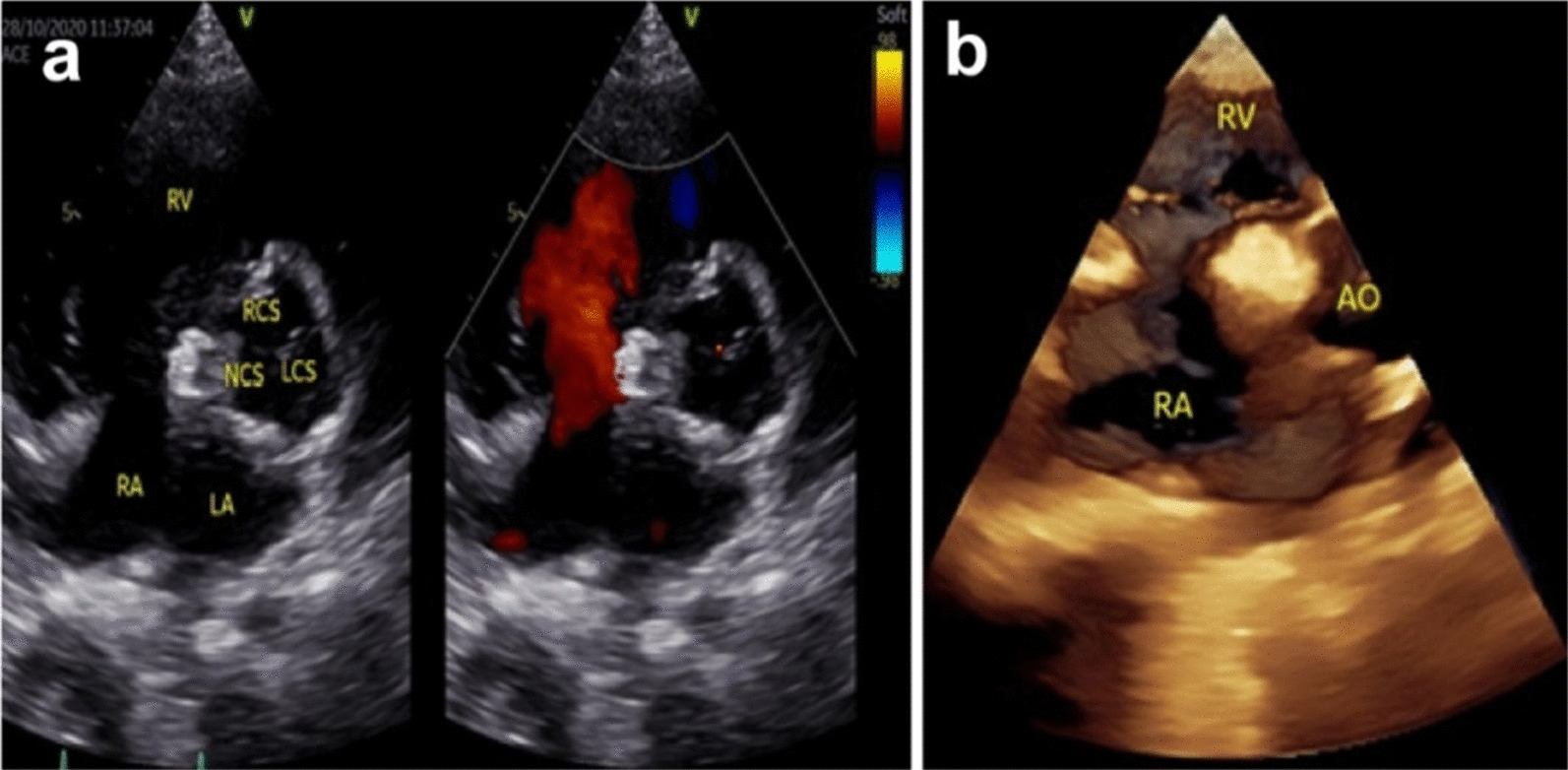
**a** Echocardiography with colour Doppler showing the closure device, and there was no shunt from the aorta to the cardiac chamber; **b** Echocardiography with a 3D imaging view of the aortic root showed the closure device. LCS, left coronary sinus; NCS, noncoronary sinus; RCS, right coronary sinus; RA: right atrium; RV, right ventricle; LA: left atrium

Cardiac catheterization and aortic artery angiography (CAG) confirmed aortic valve prolapse (right coronary valve and noncoronary valve), a noncoronary SVA that had ruptured into the RA, and an obvious shunt (Fig. 3a). Next, the patient underwent percutaneous closure intervention. The procedure was performed under general anaesthesia with CAG guidance. The pressure of the ascending aorta (AO) was 97 mm·Hg, and the pressure of the main pulmonary artery (MPA) was 34 mm·Hg, which was measured by CAG. The ruptured noncoronary SVA was measured at both the aortic end and the rupture site on angiography. The diameter at the rupture site was 7.8 mm, and a 12 mm Amplatzer duct occluder (Shanghai Shape Memory Alloy Co., Ltd, Shanghai, China) was selected for closure. The closure device could be seen clearly, and there was no shunt from the aortic valve to the RA (Fig. 3b). Postoperative echocardiography showed that there was a highlighted echo representing the closure device, and there was no shunt from the aorta to the cardiac chamber, with an LVEF of 58% and an LVFS of 31% (Fig. 4a). The reason for the postprocedural reduction in LV function may have been hyperdynamics before the percutaneous closure intervention. At the same time, the aortic dimensions were also measured, as shown in Table 1. Echocardiography with a 3D imaging view of the aortic root showed the closure device (Fig. 4b). The patient was discharged on postoperative Day 12 with an uneventful recovery.

## Discussion and conclusions

SVA, which was first reported by Edwards in 1957, can be divided into congenital or acquired [7]. The mechanism of SVA is dysplasia of sinus tissue during the embryonic period; an SVA ruptures when an acute event, such as infective endocarditis, intense activity, or some other stressor, occurs [8]. It was reported that congenital SVA is usually associated with Ehlers–Danlos syndrome, Marfan syndrome, and other tissue disorders. However, acquired SVA is frequently associated with atherosclerosis, infective endocarditis, trauma, and other factors [9]. Our patient was considered to have congenital SVA due to a lack of family history and tissue disorder screening. The patient received a genetic test for Marfan syndrome, but the result was negative.

According to the literature, SVA is usually asymptomatic in the paediatric age range and is seldom diagnosed unless it is ruptured or associated with any other severe complicated syndrome [10]. In addition, it has been reported that most SVA patients are diagnosed within a mean age range from 30 to 45 years [11]. SVA usually occurs in the right coronary sinus (approximately 70% of cases), then the noncoronary sinus (approximately 25% of cases), and finally the left coronary sinus if ruptured, and there is often rupture into the RV and RA [12]. Furthermore, Wang et al. [13] reported the same results when comparing the incidence rate of SVA between Asians and Westerners (aneurysms arising from the right coronary sinus in 86% vs. 67.8%, respectively). Therefore, our patient was diagnosed with an even rarer case of a noncoronary SVA ruptured into the RA, which is worth reporting and discussing.

The diagnosis of SVA depends on imaging tools such as echocardiography, computed tomographic angiography (CTA), magnetic resonance imaging (MRI), and CAG. Echocardiography is usually the initial diagnostic tool because of its noninvasive, low-cost, real-time, accurate evaluation of the dynamic anatomical structure, haemodynamics, and cardiac function, including the diagnosis of cardiac valve stenosis, anomaly, and valve prolapse. Regarding newer approaches, we found that CTA can show sinus origination and shunting more accurately than echocardiography can, but CTA is less helpful for intravascular blood flow assessment, is easily influenced by the heart rate and poses the risks of ionizing radiation and allergy. Magnetic resonance angiography (MRA) is a potential supplementary approach but is expensive and easily influenced by the heart rate. CAG is the gold standard for the diagnosis of SVA [14]; it can not only define the anatomy of the coronary sinus and clarify the change in haemodynamics but can also readily guide percutaneous closure intervention for SVA.

The patient in our case was diagnosed with noncoronary SVA ruptured into the RA, with a large shunt from the aorta to the RA. She was asymptomatic before being referred to the hospital with a common cold. We infer that she was affected by a congenital SVA that ruptured due to the infection. Echocardiography diagnosed the rupture of SVA first, and she received the percutaneous closure intervention immediately with good recovery. Compared to surgery, percutaneous closure intervention has the advantages of noninvasiveness and a quicker recovery with occlusive devices [15], and it may be an excellent alternative treatment for SVA. However, it should be noted that percutaneous closure intervention may also incur complications such as residual shunt, AR and TR; fortunately, after receiving the percutaneous closure intervention, our patient showed no shunt, along with mild AR and mild TR, thereby demonstrating good recovery.

In conclusion, we report a case of ruptured sinus of Valsalva aneurysm successfully treated by percutaneous closure, which is an excellent alternative treatment for SVA.

## Data Availability

The raw data could be contacted for Kunfeng Jiang who is the first author of this manuscript.

## Abbreviations

SVA: Sinus of Valsalva aneurysm
RV: Right ventricle
RA: Right atrium
LV: Left ventricle
AR: Aortic valve regurgitation
TR: Tricuspid regurgitation
PH: Pulmonary hypertension
AO: Ascending aorta
MPA: Main pulmonary artery
LVEF: Left ventricular ejection fraction
LVFS: Left ventricular fractional shortening
CTA: Computed tomographic angiography
MRI: Magnetic resonance imaging
CAG: Cardiac catheterization and angiography
MRA: Magnetic resonance angiography
